# Revealing sex-specific changes across protein structure in the aging bone extracellular matrix

**DOI:** 10.1016/j.mbplus.2025.100189

**Published:** 2025-12-25

**Authors:** Jacob Tudor, Alexander Eckersley, Michael Buckley

**Affiliations:** a School of Biological Sciences, Faculty of Biology, Medicine and Health, The University of Manchester, Oxford Rd, Manchester M13 9PL, UK; b Manchester Institute of Biotechnology, School of Natural Sciences, Faculty of Science & Engineering, The University of Manchester, 131 Princess Street, Manchester M1 7DN, UK

**Keywords:** Peptide location fingerprinting, Rat, Bone, Aging, Proteomics

## Abstract

The process of aging is an integral but complex component of life that has been intensely studied for decades, from the molecular level to whole organisms. At the tissue level, bone is one of the most difficult to study due to its composite nature of inorganic and organic phases, but advancements in proteomics are enhancing our understanding of the latter, improving our understanding of skeletal aging through identifying temporal changes across the bone proteome. The relative longevity of extracellular matrix (ECM) proteins can make them more susceptible to accumulating damage modifications over time. In addition, their informational density, including their 2D and 3D structure, protein folding, post translational modifications and proteomic composition, as well as their functional importance, has made them a target of interest in the study of aging and medical conditions such as osteoporosis and arthritis. ECM proteins are also increasingly utilised in forensic science for determining biological sex/age because of their longevity. Following recent developments in peptide location fingerprinting methods that improve capabilities of identifying regional changes in protein structure, this study aimed to identify age-associated regional changes along protein structures in *Rattus norvegicus* from whole limb LC-MC/MS data. Regional changes in protein structure were identified in a variety of collagenous and non-collagenous ECM proteins, providing evidence for increased remodeling in juvenile rats and a reduced ability in adult rats, alongside damage accumulation in the adult ECM. This research highlights the importance of fibrillar collagen remodeling but is also indicative of potential new roles for osteopontin, thrombin, apolipoproteins and wider ECM regulators such as cartilage oligomeric matrix protein. This demonstrates, in this case, the utility of peptide location fingerprinting as a screening tool to identify biomarker candidates of bone aging between juvenile and adult rats.

## Highlights

• First use of peptide location fingerprinting as a screening tool to identify biomarker candidates of bone ageing.
• Highlights importance of fibrillar collagen remodelling.
• Suggests new roles osteopontin, thrombin, apolipoproteins and wider extracellular matrix regulators.

## Introduction

Our understanding of aging has expanded with the increased use and development of proteomic technologies, in combination with innovative computational methods of data analysis. Identifying proteomic changes in relation to aging is now well established, with protein abundance and composition showing variance in a range of tissues [1], [2], [3]. However, the study of aging in osseous tissues is of notable interest considering the onset of age-related degradation in bone and the incidence of degenerative diseases such as osteoporosis [4]. As bones age, they become increasingly fragile, prone to breaks and slow to repair [5]. Therefore, an increased understanding of age-associated proteomic changes in bone could improve efforts for reversing or preventing the onset of age-related bone disease and degradation.

Obtaining biological insights from bone tissue is highly valuable due its inherent longevity, being the last tissue to decompose after death. Furthermore, the bone proteome maintains its integrity better than other biological material such as DNA. This has made the study of bone proteomes increasingly important in expanding our understanding of aging and age-associated pathology. In a related line of research, proteomic studies have also become valuable tools in forensics, providing insights able to assist in the determination in causes of death [6]. Furthermore, the detection of biological age- and sex-related proteomic changes is becoming more routine in several mineralised tissues [7], [8], [9], such as with the use of tooth enamel in the determination of sex in by assessing abundance of sex-dependent amelogenin protein with high success rates in forensic science [10] as well as archaeological remains [11], [12]. However, these approaches have been less studied in bone, not yielding sex-linked protein differences (i.e., primary structure/protein sequences), despite it being a much more abundant tissue type. In forensics, age- and sex-determining methods still rely on differentiating morphological/anthropological characteristics and have been shown to greatly rely on individual experience of the forensic specialists where the determination of age is still subject to error in the region of decades [13]. Hence there is the need for an alternative approach, less subject to individual observer bias, with proteomic methods potentially able to provide that alternative complementary approach.

The prospect of utilising proteomics for the determination of biological age and sex in animal models has been previously explored [1], [3], within which significant differences have been seen in the relative abundances of particular proteins. Using subsampling on specific parts of different skeletal elements, Procopio et al. [3] identified a select number of serum proteins as markers, whereas in whole limb LC-MS/MS proteome data, Johnston & Buckley [1] widened the range of proposed markers; the latter study was furthered to also investigate changes in the relative amounts of post-translational modifications (PTMs) in rats of varying age groups [14]. As tissues age, protein structures alter through PTMs, seen in the quick development of juveniles to adulthood [15], [16], and the accumulation of biological damage to protein structures and the networks regulating their synthesis [17], [18]. This can be seen particularly in long-lived proteins of the extracellular matrix (ECM) such as the collagenous proteins making up the majority (∼90 %)[19] of the organic ECM in bone tissue. Due to the relatively long half-lives of ECM proteins [20], [21], [22] such as structural collagens, they become more vulnerable to the accumulation of damage modification from such events as the addition of advanced glycation end products and oxidative stress [23], [24].

It is well established that protein structural changes can occur through mutation, alternate splicing, and PTMs. This can cause fundamental changes to protein folding, with alterations to secondary structure, subsequently modifying or preventing tertiary and quaternary protein folding, ultimately changing or inhibiting protein function. The location of such modifications is equally important to the functional integrity of the protein, where modifications to active and co-active sites as well as to binding regions have some of the largest impacts on protein function. For example, PTMs including covalent additions to protein structures, can significantly alter protein folding and function [25] such as oxidation (lysine, proline, methionine), deamidation (asparagine, and to a lesser extent glutamine), glycosylation, and fragmentation by proteolytic cleavage. Numerous studies involving the analysis of PTMs have previously shown their accumulation in the proteins of a wide range of aging tissues [14], [26], [27], [28] and that protein structures themselves change with age [29]. The proteo-stasis network (dynamic regulation of a functional proteome) becomes more susceptible to mutation, misfolding and accumulation of post translational modifications leading to dysfunctional protein structures [30], [31]; this is paired with a reduced ability of correctional machinery (such as heat shock protein 70 kDa (HSP70)[24], [32]) and pathways to deal with this misfolding. The degradation of the proteo-stasis network as such has become a major hallmark of aging, but understanding the dynamics of proteo-stasis dysregulation in relation to the ECM remains an understudied field in aging research [24] that warrants further exploration, particularly with regards the effects of regional age-associated changes in protein structure.

Although the unbiased identification of abundance-independent proteomic markers like PTMs is difficult, peptide location fingerprinting (PLF) is a novel bioinformatic approach with the potential to illuminate further such biomarkers. PLF utilises LC-MS/MS-derived datasets to map and statistically quantify tryptic peptides across protein structures, identifying regional differences in trypsin digestibility that are indicative of modifications. This is not to be confused with peptide mass fingerprinting (PMF), a cruder method developed prior to LC-MS/MS analyses that involves the mapping of observed peptide precursor *m*/*z* values against theoretical values of protein sequences digested *in silico*. LC-MS/MS analyses nonetheless improved interpretations of PMF, affording the ability to identify peptides more confidently. However, PLF has the advantage of incorporating relative abundance information from LC-MS/MS datasets. PLF was initially used to detect photo-induced damage to ECM protein structures *in-vitro* by ultraviolet radiation, unveiling conserved cumulative patterns of protein susceptibility to tryptic cleavage across two intensities via broadband UVB and solar simulated radiation [33]. This demonstrated the possibility that PLF could be applied to studying age-associated damage to protein structures. PLF has since identified age-related differences in ECM protein structure in kidney, lung, intervertebral disc (IVD) and tendon tissues using historical, publicly available datasets [34], [35] and proven useful as a screening tool for identifying modified ECM protein modifications independent of protein abundance. For example, conserved differences in peptide yield were previously identified across the collagen α2(I; COL1A2, hereafter gene names used after introduction of protein names) protein structure in three distinct IVD regions [34]. These findings revealed elevated collagen (I) synthesis in younger samples, indicated by a higher presence of C-terminal propeptides, and increased matrix metalloproteinase (MMP)-mediated degradation in aged samples, evidenced by greater peptide yields downstream of a prominent MMP cleavage site. This demonstrates the capability of PLF to detect multiple aging-related mechanisms and consequences within individual proteins *in vivo*. More recently, structural alterations in basement membrane proteins periostin and collagen α2(IV; COL4A2) were observed, with consistent aging-related patterns across human kidney and mouse lung [35]. For COL4A2, significantly lower peptide yields were detected in aged samples within the globular NC1 domain, a region known to be susceptible to MMP cleavage that releases the bioactive matrikine canstatin. This approach can provide a foundation for follow-up study of their effect on protein function and the nature of the modification and, in tandem with abundance-driven biomarker discovery, can help to provide another layer of depth to the inferred characterisation of PTMs in LC-MS/MS data.

This study aims to analyse datasets previously published [1] using PLF, with the goal of identifying structure-associated, biomarker candidates of aging bone. In doing so providing a new depth of structural analysis to biomarkers previously identified through protein quantitation and the identification of new biomarkers through regional differences in peptide level quantitation, providing insights into age-associated changes in protein structure. Here we focus on male and female specimens of *Rattus norvegicus* juvenile (1–2 weeks old) and adult (10 weeks − 6 months old) age groups. We hypothesise that ECM proteins will exhibit regional differences in peptide yield across protein sequence structure in adult compared to juvenile tissues, indicating age-related modifications associated with bone remodeling.

## Methods

### Data generation

The raw mass spectrometry and peak list data were obtained from the ProteomeXchange Consortium PRIDE repository under dataset identifier PXD022055 [1]. The quantitative mass spectrometry data was derived from whole *Rattus norvegicus* fused tibiae and fibulae. This study used 12 samples from the previously published dataset to compare the youngest age group, 6 x juvenile rats (age 1–2 weeks) and the oldest age group, 6 x adult rats (age 10 weeks − 6 months) with equal data representation in both sexes.

MS/MS ion searches were on peak list files performed using MASCOT v 2.5.1 (Matrix Science, MA, USA). Searches were performed against the SwissProt database (2018) using Mascot v2.5.1 with the following parameters: taxonomy: *Rattus*; number of proteins (8036); fragment tolerance: 0.5 Da; parent tolerance: 5 ppm; fixed modifications + 57 on cysteine (carbamidomethylation); variables modifications: +1 on asparagine and glutamine (deamidation), +16 on lysine, methionine, and proline (oxidation); digestion enzyme: trypsin; maximum missed cleavages: 1; peptide charge 2+, 3+, & 4+. These were carried out for each sample group separately and the results exported as both .dat for uploading into Scaffold 5 software (Proteome Software, OR, USA), and .csv files for analysis of specific PTMs (see supplementary material S3). The .dat files were uploaded into Scaffold where protein and peptide identification probabilities were calculated using the Trans-Proteomic Pipeline and the PeptideProphet™ algorithm this was thresholded to ≥ 95 %. This resulted in a low false discovery rate of 0.5 % and a greatly reduced chance of proteins being identified by false positives. Peptide lists of proteotypic peptides and their exclusive spectral counts were exported from Scaffold. Anaconda v2.5.0, Jupyter-notebook v6.5.4, Python 3 v3.11.5 in combination with Pandas v2.0.3 was used to format the data to make it compatible for analysis by the MPLF webtool as previously described [36]. Per sample protein total peptide spectral counts can be found in Supplementary Table S2 for unfiltered data and Supplementary Table S3 for the Scaffold filtered data for proteins further explored in this study.

**Fig. 1:**
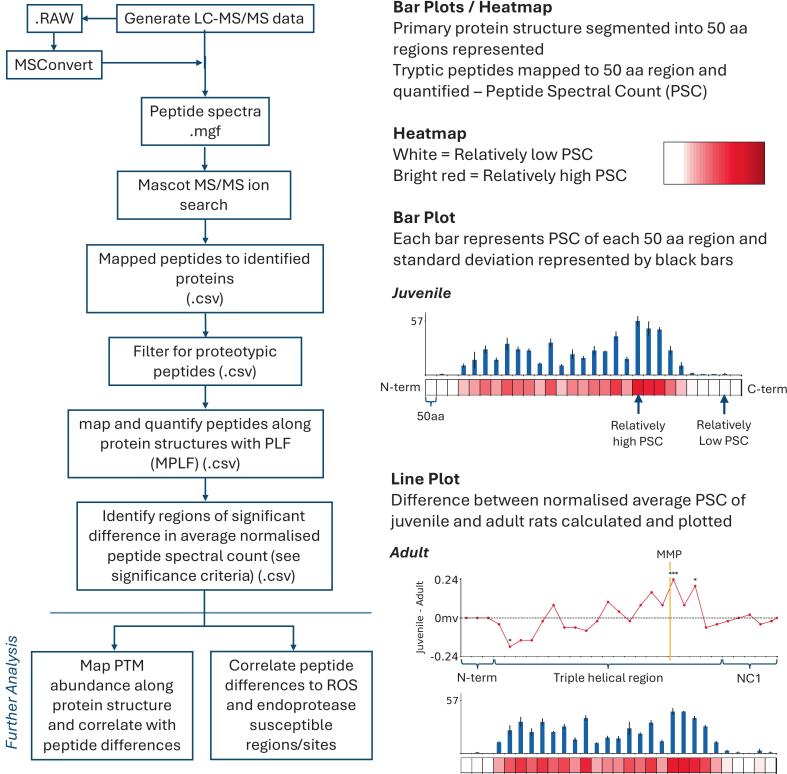
A) Flowchart of data (peptide list and protein search) generation using Mascot, processing and analysis of peptide spectral counts using the Manchester Peptide Location Fingerprinting tool (MPLF). Mascot peak spectra files (.mgf) of LC-MS/MS data from the ProteomeXchange Consortium PRIDE repository (dataset identifier PXD022055) was used to perform MS/MS ion search against the SwissProt database, using Mascot Server and Mascot Daemon to generate.dat files, which were imported into Scaffold 5 and filtered to proteotypic peptides and false discovery rate of 0.5 %. PLF analysis was run on the six juvenile and six adult rat specimens with equal data representation of both sexes. Results from males and females were overlayed to assess sex specific differences. B) Breakdown of COL1A1 PLF data visualisation. Bar plot represents tryptic peptides mapped to each 50aa region (peptide spectral count; PSC) of COL1A1 primary structure and the standard deviation for each region. Heatmap shows range of PSC across the structure with bright red representing a 50aa region with a high PSC. Line plot also presents differences in average normalised PSC (Y-axis) between juvenile and adult rats at each 50aa region (X-axis) in COL1A1 with important structures and domains labelled below. Important aa sites like cleavage sites displayed using yellow lines. 50aa regions showing statistically significant age-associated differences (repeated measures Anova with Bonferroni correction) shown using * (* = p ≤ 0.05, ** = p ≤ 0.01, *** = p ≤ 0.001). *Note that this methodology and data pipeline can be translated to other mass spectrometry bioinformatics tools such as MSFragger; PLF requires peptide list CSV files and the corresponding spectra generated from MS/MS protein searches. (For interpretation of the references to colour in this figure legend, the reader is referred to the web version of this article.)

PLF analysis compared male and female aging datasets separately with identified proteins from the SwissProt database segmented into 50 amino acid (aa) regions and the peptides mapped to each region (Fig. 1A) to provide an optimal standardised aa range for analysis across the entire protein structure of both large and small proteins consistent with prior studies utilising PLF [33], [34], [35], [36]. Peptide spectral counts were summed for each 50aa protein region, median normalised based on total protein spectral count (to remove skewing caused by differences in whole protein abundances) and statistically compared between age groups using a Bonferroni-corrected two-way repeated measures ANOVA to identify structural differences.

### Visualisation of PLF results

Graphical representation of the analysis data was created to visualise and interpret PLF results and facilitate the identification of significant differences in peptide yields across protein structures. These were made using Anaconda v2.5.0, JupyterNotebook v6.5.4, Python3 v3.11.5, Pandas v2.0.3, Numpy v1.24.3, Matplotlib v3.7.2. Average normalised regional peptide spectral counts (PSC) were visualised using bar plots per 50aa region and a heatmap was used to indicate relative average PSC abundance and coverage across each protein (e.g., Fig. 1B).

The difference in average median normalised PSC, relative to each 50aa segment, between juvenile and adult rats was taken (i.e., juvenile – adult/50) and plotted for males and females. Significance levels were represented by “*” identifiers. p < 0.05 = *, p < 0.01 = ** and p < 0.001 = ***, based on Bonferroni-corrected two-way repeated measures ANOVA.

### Identifying regions of significant age-associated difference in proteins

To reduce the chance of evaluating false positive changes and to ensure only the most robust age-associated biomarker candidates were identified, 50aa protein regions were designated as significant if they had Bonferroni corrected repeated measures two-way ANOVA significances of p ≤ 0.05 across the 3 specimens per age group, and a minimum, average PSC difference ≥ 2 peptides. This designation also required the 50aa regions to have a minimum average normalised PSC of 1 in both compared groups, to prevent significance being attributed to missing values in one group only.

Proteins were considered to contain significant regional differences even with poor total protein coverage. Total percentage protein coverage was also plotted for each of the proteins identified as containing significant regions of age-associated structural difference for all data groups (Supplementary Fig. S1). Additionally, for all comparisons of PSC, protein differences reported were those detected in all samples relevant to the indicated grouping of males, females, or both (i.e., if protein matches were not observed in one sample of one of the sexes, then it would only be reported for the other sex; Supplementary Tables S2, S3). This criterion keeps biological interpretation of regional differences grounded, highlighting the challenge of obtaining optimal peptide identifications in complex tissues from tandem mass spectrometry methods.

### Global assessment and protein class identification

Protein biomarker candidates identified with the above criteria were presented in Venn diagrams with those identified to have age-associated changes in abundance from Johnson and Buckley [1], comparing males with females. Male-unique, female-unique and shared biomarker candidates were run through Panther 19.0 [37] for protein class and biological function determination [38] and visualised using pie charts.

## Identifying impacts of age-associated regional differences

All regions considered to be significantly different between age groups were correlated with MS-identified average (median) PTM counts subject to median normalisation against total protein abundance (as done for PSC; see Supplementary Figs. S2 & S6), SwissProt database and relevant published literature describing the structure and functional regions of each protein. Notable annotated regions and regions of interaction such as active and co-active sites were noted, as were regions of possible importance to protein folding or bridging regions. Reactive oxygen species (ROS) and matrix metalloproteinase (MMP) cleavage susceptibility in human proteins, derived from the Manchester Proteome Susceptibility Calculator (MPSC) tool [36] was also correlated.

## Results and discussion

### Extracellular matrix is the main class of proteins harbouring structure-associated differences between young and aged.

**Fig. 2:**
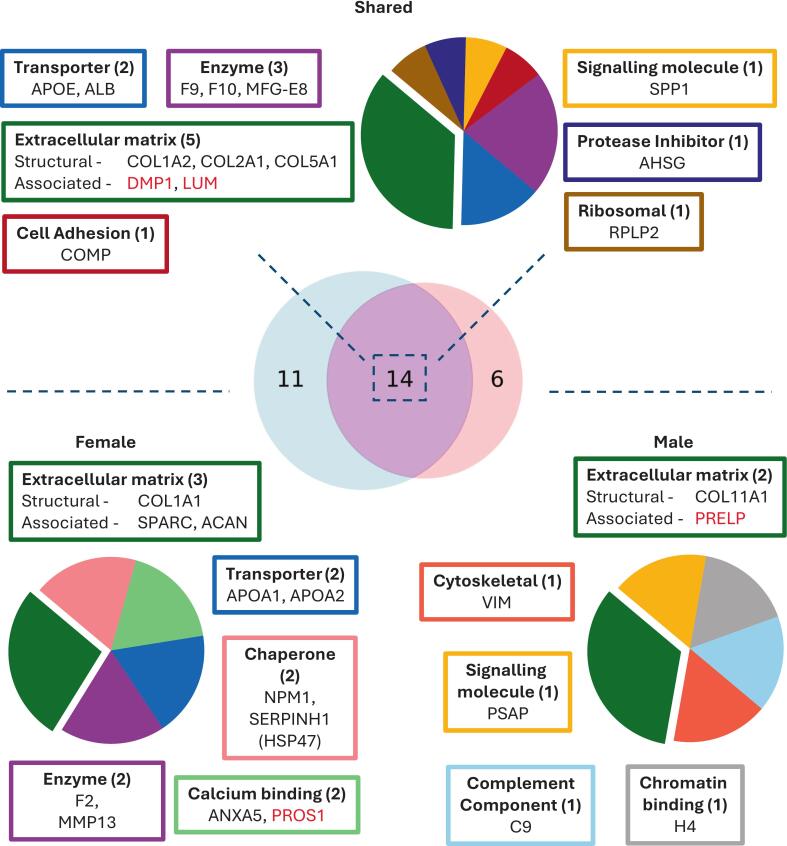
Protein classes for all biomarker candidate proteins considered to have at least one significant regional difference between juvenile (1–2 weeks) and adult (10 weeks – 6 months) rats. The Venn diagram displays the number of candidates identified to have significant age-associated differences in the either the female or male groups only, or both. Classes were identified using PantherDB and further grouped based upon their prominent features defined in their UniProt entries. The ECM glycoprotein class was grouped into an ECM-associated group. Serine proteases, metalloproteases and oxidoreductase classes have been grouped as enzymes, scaffold adaptor protein and cytokine classes have been grouped as signalling molecules. Proteins with no assigned PantherDB class (red) have been placed into a representative class based upon their prominent biological processes within their UniProt entries. Pie plots present relative proportion of classes represented in proteins found to have age-associated differences. (For interpretation of the references to colour in this figure legend, the reader is referred to the web version of this article.)

The Mascot MS/MS ion search (summarised in Supplementary Table S1) resulted in 1640 unique tryptic peptides that were mapped to 147 proteins from both male and female datasets. 1287 tryptic peptides mapped to 109 proteins from the male rat data and 1174 tryptic peptides mapped to 100 proteins from the female rat dataset. Of the 31 proteins that met the criteria as biomarker candidates, the dominant class of proteins were structural ECM proteins (Fig. 2; Table 1). The biomarker candidates uniquely identified in female rats shared dominant protein classes of ECM proteins and apolipoproteins. Of the six candidates uniquely identified in male rats, each belonged to a unique protein class.

**Table 1:** Summary of key findings and interpretations indicated by significant changes in regional peptide spectral counts (PSC) within structural and regulatory extracellular matrix proteins in rat bone.

Protein	Regions	Region Significance	Explanation for change in PSC	Interpretation
APOE	aa200-300 (male and female)	Contains VLDL and Lipid association domains	Increased cleavage of APOE by MMPs creates more soluble fragments and greater accessibility to the action of trypsin	Cleavage of APOE indicator of dysfunctionality which could lead to an accumulation of lipids within bone extracellular matrix
COL1A1	aa950-1000 & aa1050-1100 (female)	C-terminal domain within close proximity to known MMP cleavage sites	Increased MMP action creates more soluble fragments and greater accessibility to the action of trypsin	Increased MMP cleavage indicates increased degradation of mature collagen fibres in juvenile rats
COL1A2	aa1000-1100 (male and female)	C-terminal domain within close proximity to known MMP cleavage sites	Increased MMP action creates more soluble fragments and greater accessibility to the action of trypsin	Increased MMP cleavage indicates increased degradation of mature collagen fibres in juvenile rats
COL2A1	aa1150-1250 and aa1300-1400 (NC1 domain) (male and female)	Contains NC1 domain of pre-Col2a1	Increased presence of NC1 domain in adult rats	The NC1 domain of pre-collagen is cleaved during the synthesis of collagen prior to trimerization. Detecting NC1 domain peptides would indicates active collagen synthesis and significantly greater Col2a1 synthesis in adult rats.
COMP	aa600-650 (male and female)	Contains region that mediates cell survival in chondrocytes	No direct evidence of increased cleavage of Comp, Age-associated changes to structural conformation in adults resulting in increased accessibility to trypsin	Increased susceptibility to cleavage in adult rats could indicate reduced viability for comp to mediate cell survival in chondrocytes
SPP1	aa150-200 (male and female)	Contains F2 cleavage site that activates SPP1	Increased cleavage of SPP1 by F2 creates more soluble fragments and greater accessibility to the action of trypsin	Cleavage of F2 cleavage site activates SPP1 indicating a greater presence of active SPP1 in juvenile rats and subsequently greater remodelling
F2	aa359–360 (female)	Contains Xa cleavage site that activates F2	Increased cleavage of Xa site by F2 creates more soluble fragments and greater accessibility accessible to the action of trypsin	Cleavage of Xa site activates F2 indicating a greater presence of active F2 in juvenile rats

It was expected that the primary group of proteins would be related to ECM structure or maintenance due to its large representation (e.g., collagens) within bone tissue [19]; this was consistent in both male and female specimens. Identification of age-associated changes to protein structure in collagenous proteins is likely due to their long half-lives and thus increased vulnerability to damage accumulation over time [19], [24].

Of the structure-associated biomarker candidate proteins identified by PLF (Supplementary Fig. S2), 25 proteins were not previously identified as potential biomarkers of aging in rats based on relative protein abundance. Of the nine proteins previously found in rat bone by Johnston and Buckley to be significantly associated with age based on abundance changes [1], three proteins, PGS1, Chromogranin-A and Matrilin-1 were not detected to have any structural age-related protein differences by PLF whereas the other six proteins also harboured significant regional differences in protein structure.

The results of PLF generated by the MPLF tool showed 31 of the 147 identified proteins as displaying significant age-related regional differences in protein structure. Of these, osteopontin, albumin and collagen α2(I) showed excellent protein sequence coverage in juvenile and adult rats for male and female specimens (Supplementary Fig. S1). Over 50 % of the proteins had coverages of > 40 % in at least juvenile or adult rats. Similar protein sequence coverage was observed in adult and juvenile rats with notable differences being seen in F9, F10, DMP1 in both male and female rats. Across the seven proteins explored further in this study five showed consistent patterns in regional peptide spectral counts conserved across sexes. This indicates signs of sex-conserved differences and providing evidence that the changes observed were likely not to be the result of false positives.

### Aged fibrillar collagens exhibit structure-associated differences in protein regions associated with fibrillogenesis and proteolysis

**Fig. 3:**
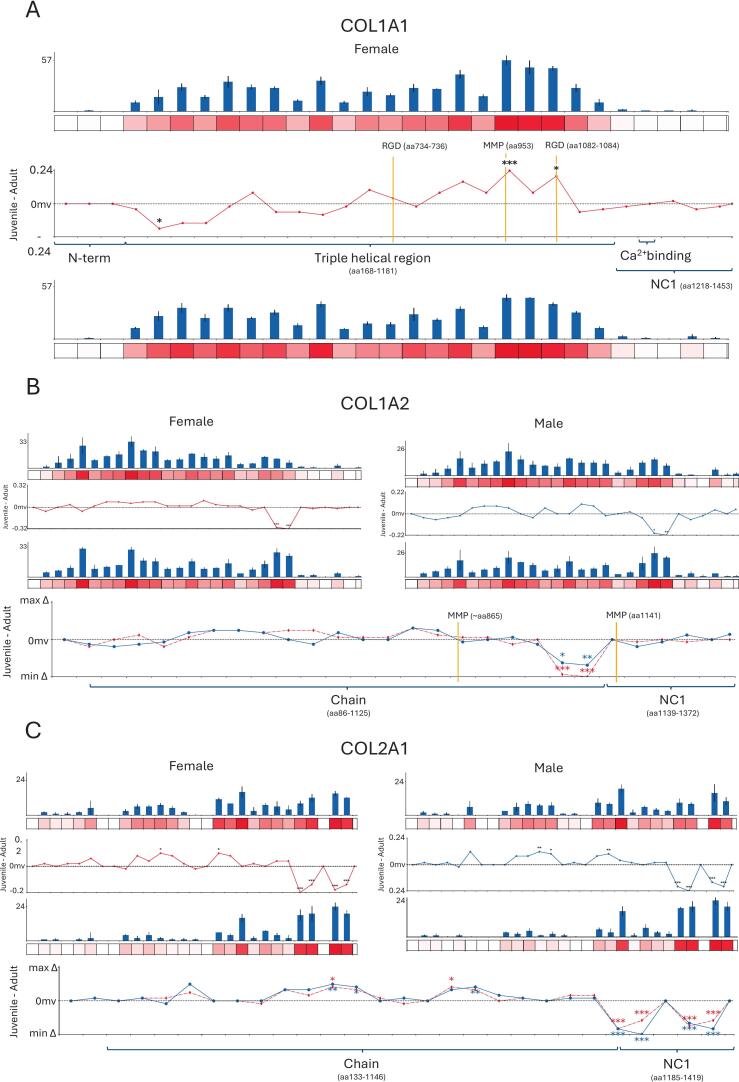
Collagenous proteins with 50aa regions exhibiting age-associated differences in average normalised PSC. Bar plots represent average normalised PSC per 50aa region, heatmap below represents the relative PSC across the protein, bright red representing a high relative PSC. Line plots show the difference in normalised average PSC (Δavg.PSC) plotted (Δavg.PSC in juvenile rats [1–2 weeks old] − Δavg.PSC adult [10 weeks – 6 months old]) against the quantified 50aa regions. Levels of significance are indicated by p < 0.05 = *, p < 0.01 = **, p < 0.001 = ***. Published cleavage sites are indicated with a yellow line. A) Three regions of significant differences were identified in COL1A1 in female rats. One just downstream of N-terminal propeptide and two upstream of NC1 domain. B) COL1A2 had two regions upstream of the NC1 domain with significantly higher PSCs in aged compared to young, identified in both male and female groups. C) Male and female COL2A1 also harboured four regions within the NC1 domain with significantly higher PSCs in aged compared to young. (For interpretation of the references to colour in this figure legend, the reader is referred to the web version of this article.)

Ten of the 31 biomarker candidate proteins identified with statistically significant age-related differences were ECM proteins (Fig. 2). Of the ten, five were fibril-forming collagens. Fibril forming collagen chains α1(I; COL1A1) and α2(I), α1(II; COL2A1), α1(V; COL5A1 and α1(XI; COL11A1) all showed statistically significant regional age-associated differences across their protein structures. Of these, COL1A2, COL2A1 and COL5A1 exhibited statistical differences in both male and female rats. Due to the relatively lower coverage of COL5A1, only COL1A1, COL1A2, and COL2A1 are investigated in more depth (Fig. 3).

Some protein regions of significant difference identified in COL1A1 and COL1A2 were found at the C-terminal end of the mature triple helical chain, where important matrix metalloprotease (MMP) cleavage sites are also located (Fig. 3A & C). A cluster of significantly age-affected regions were also identified within the NC1 C-terminal pro-peptide domain of COL2A1. This domain is cleaved off from pro-collagen during the synthesis of fibrillar collagen by C-proteinases, like bone metalloproteinase 1 (BMP-1) [39], [40], [41]. Observations of increased difference in PSC within the NC1 domain itself would be an indicator of increased collagen synthesis, however differences upstream of the NC1 domain, within the mature fibre, close to known or predicted MMP cleavage sites would be an indicator of increased collagen degradation [35], [42], [43].

Collagen (I) is a heterotrimeric protein comprised of two α1(I) chains and one α2(I) chain. Three 50aa regions were detected as having significant regional age-associated changes in PSC in COL1A1 in female rats. Two of the regions (aa950–1000 and aa1050-1100) showed significantly higher PSCs in younger rats upstream of the NC1 domain, within the mature triple helical region. These lie near to published MMP1, 2, 9, 13 and 14 cleavage sites (identified by MPSC) [36] and could indicate increased proteolytic degradation of collagen I by a variety of MMP cleavage activity. Initial cleavage of collagen tends to occur by the action of MMP1, 8, 13, 14, 16, and possibly MMP15 to produce archetypal 3/4 and 1/4 fragments observed from collagenolysis [44]. Further degradation of collagen then occurs from subsequent MMP2 and 9 cleavages [45].

The presence of regional differences in peptide yield at the COL1A1 RGD (aa1082-1084) (Arg-Gly-Asp) cell attachment motif also could be significant for the integrin binding of osteoblasts, important for promoting bone remodeling and repair [46]. Previous research indicates these motifs becoming more exposed with the accumulation of damage or denaturing of collagen I fibres [47]. The significantly higher PSCs seen in COL1A1 of younger rats could indicate a change in cell attachment which would have implication for bone remodeling. The regional differences in PSCs are also located upstream from the Ca^2+^ binding domains found in the C-terminal end of the COL1A1 triple helical chain. Since these differences are an indicator of reduced trypsin cleavage in older rats, this could indicate a reduction in bioavailability of the Ca^2+^ binding domains, impacting on hydroxyapatite crystal formation needed for collagen I maturation and potentially bone fragility in adult rats [15]. Interestingly however, across COL1A1 we observe 19 peptide sequences that are unique to adult samples whereas only 4 that are unique to juvenile specimens. This potentially indicates a wider variety of cleavage sites in adult rats compared to juvenile rats, perhaps due to the accumulation of damage. However, PTM analysis of oxidised methionine accumulation within the regions of significant PSC difference, did not indicate damage due to ROS accumulation. It is more likely that the differences we see in PSC are attributable to an increased activity of MMPs cleaving COL1A1 creating more soluble fragments more bioavailable to trypsin activity.

Similar regional differences were observed in COL1A2 (Fig. 3C) and COL2A1 (Fig. 3D) to what was seen in COL1A1, with significantly higher PSCs identified near the C-terminal ends the adults for both males and females. In COL1A2, the two adjacent regions aa1000-1050 and aa1050-1100 are located downstream of published MMP, 1, 2, 13 and 14 cleavage sites (∼aa865), with region aa1050-1100 being immediately upstream of a published MMP-9 cleavage site (aa1141). PTM analysis also indicated greater deamidation and an increase in the median number of hydroxylated prolines in adult rats (Supplementary Fig. S5) perhaps indicating a greater maturation in collagen (I) in adult compared to juvenile rats. A lack of significant average accumulation of methionine oxidation across all protein specific peptide reads provides no direct indication of ROS accumulation suspected by MPSC (Supplementary Fig. S3). Similarly, evidence of increased cleavage upstream of the NC1 domain suggests increased rates of collagen degradation and collagenolysis in adult rats.

Interestingly, structure-associated differences were present in regions preceding the NC1 domains of both COL1A1 and COL1A2, manifesting as lower PSC abundance in adult rats for COL1A1 and higher for COL1A2. Due to these regions lying downstream of prominent MMP cleavage sites, this could be evidence of changes in collagen (I) remodeling in the developing juvenile compared to the mature adult. Juvenile rats have high bone remodeling during growth and development compared to mature adults where collagen undergoes cyclic maintenance and circadian homeostasis [48], [49], [50]. Differences in these regions were conserved between males and females, indicating that this remodeling is conserved between sexes. Uniquely to COL1A2, a large disparity of peptide numbers was observed, with 41 peptides that were uniquely identified in adult compared to 10 in juvenile samples (Supplementary Table S4). This could indicate again a more selective regional form of tryptic cleavage indicative of a greater presence of deposited fibrillar collagen (Supplementary Table S4). Simultaneously regions aa1000-1050 and aa1050-1100 contained peptides that were on average longer than those in juveniles, with region aa1050-1100 showing a median peptide length approximately twice the length (Supplementary Fig. S4), providing evidence for the higher permanence of adult collagen while also suggesting adult collagen could be cleaved in larger peptide lengths. These factors contribute as indicators of a greater accumulation of damage across COL1A2, which is regionally supported by the increase in median proline hydroxylation count alongside deamidated asparagine and glutamine found within peptides mapped to regions spanning aa1000-1100 in adult male and female rats suggesting increased maturity of collagen fibres in adult rats. Despite no direct indicator of ROS accumulation in the form of significant increases in oxidised methionine. Evidence of increased PSC abundance was previously observed within the same 50aa region, upstream of the NC1 domain, of COL1A2 in old human intervertebral disc (IVD) compared to young [34], indicating that age-dependant collagen remodeling likely occurs in osseous tissues of both humans and rats. These increases in peptide spectral counts could be attributed to increased MMP cleavage resulting in an increase in more soluble fragments accessible to trypsin causing the increase in tryptic peptide observed. Higher PSC abundance was also observed within the C-terminal propeptide (NC1 domain) of COL1A2 in young human IVD [34], indicating higher collagen I fibrillogenesis. Further studies into the turnover of COL1A2 and its reparation mechanisms compared to COL1A1 are needed with respect to rat bone as we see no evidence of differences within the C-terminal propeptide in this study.

Six COL2A1 50aa regions in females, and seven in males, harboured statistically significant PSC differences in juvenile compared to adult rats (Fig. 3D). Two of these regions in female and three in male were found in the triple helical domain of and had higher PSC abundance in young rats. The other four 50aa regions showed significantly higher PSCs within the NC1 domain of adult rats, which was conserved in both males and females.

Collagen (II) is comprised of a homotrimer helix of α1(II) chains and is the primary component of articular cartilage ECM. Type II cartilage is present in the epiphyseal growth plates of bones and undergoes constant remodeling that takes place even in adulthood, but to a lesser extent than in developing juveniles [51]. MMPs 1, 2, 9, 13, and 14 cleavage sites are located throughout the triple helical region (e.g. region aa800-850 contains a cleavage site at aa834–83) [52], so higher PSC abundances within this may indicate more collagen (II) degradation in the juvenile groups. Greater PSCs in the triple helical regions could again be attributed to the increased remodeling found in juvenile skeletons. Interestingly, significantly higher PSC abundance was identified within the NC1 domain of both male and female adult rats, which indicates increased fibrillogenesis of collagen (II) via the greater presence tryptic peptides belonging to the cleaved C-terminal of procollagen [53]; a region only present during collagen synthesis before being cleaved post collagen trimerization. This includes the 50aa region with the C-proteinase cleavage site at aa1241–1242 [52] in older rats. This is unexpected, as mammalian adult skeletons mature in adulthood and collagen (II) synthesis tends to reduce in comparison to juveniles [54]. Very similar observations were made previously in COL2A1 of young human IVD, which also had NC1 domain-specific, higher PSCs in young than in aged, indicating that collagen (II) synthesis is lower in older individuals [34]. We observe different but significant indicators of remodeling occurring in juvenile and adult rats; this reflects the reality of constant remodeling into adulthood through proteolytic degradation and synthesis [51]. Of note on the susceptibility of COL2A1 to tryptic cleavage, is the high but relatively even number of unique peptide sequences identified to be contributing to PSC (Supplementary Table S4) specifically in female rats with equally 11 peptides uniquely identified in juvenile and adult female rats. In male rats, 22 were uniquely identified in juvenile with only 6 being unique to adult rats. This sex specific increase in identification of unique peptide sequences in juvenile male rat samples overall seems to point to an increase across the breadth of the protein in cleavage susceptibility and it is interesting that we see this in samples indicating a higher turnover of COL2A1 perhaps indicating the temporary nature of this protein in a high turnover environment seen in juvenile bone remodeling. Counter to this is the even greater expected turnover from adult rats in this study due to the significantly greater abundance in PSC within the NC1 domain.

It would be crucial to explore the cleavability afforded by confirmation *in-vivo*/*vitro* experiments to validate the impact of PTMs on tryptic accessibility. Overall, there was an increase in median hydroxylated proline, oxidised lysine and deamidation abundance across adult Collagen supporting the assumption of increased maturity of collagen fibres in adult bone tissue. While PTM analysis of hydroxylysine accumulation did not reveal any significant insights to infer our observed changes in PSCs future analyses may benefit from addressing modifications to arginine which can have direct impacts on tryptic cleavage. Despite this, since MS/MS loses detection sensitivity with high charge states and high *m*/*z* features it optimally identifies peptides between ∼6 and ∼25 amino acids and the sporadic distribution of lysine and arginine across collagen, there are only a small number of cases as seen in prior studies of collagens [55] where the singular missed cleavage site would not be adequate in addressing both lysine and arginine modifications.

In our analysis the low identification frequency of peptides assigned to SerpinH1/HSP47 during our protein search made it unfeasible to draw any insights from the statistically significant increase in PSC abundance within the 50aa region housing the endoplasmic reticulum retention signal in female juvenile rats. The premature loss of this signal would result in increased lateral aggregation of misfolded collagen and the degradation of the ECM [56]. The increased identification of this signal in juvenile rats could indicate a stronger presence of HSP47 localised to the endoplasmic reticulum, contributing to proper procollagen maturation however it is impossible to determine from this data whether reduced identification of the ER retention signal is premature or a snapshot into the release of mature procollagen from the endoplasmic reticulum to the Golgi apparatus [57]. Future studies aiming to identify age-associated changes within HSP47 could provide crucial insights into the repair/remodeling of the ECM.

### Osteopontin and prothrombin exhibit age-specific differences in protein regions critical for functional activation

Osteopontin (bone sialoprotein 1, SPP1) is a major ECM matrix regulating protein with multiple functional roles in cellular migration, cellular attachment, activation of the immune system and tissue remodeling [58] where much of this functionality comes from cellular adhesion through RGD integrin, heparin and Ca^2+^ binding domains. Activation of SPP1 typically requires the cleavage of a conserved thrombin cleaving domain which gives rise to an N-terminal peptide containing a more exposed RGD integrin binding motif; this cleavage also gives rise to a C-terminal peptide containing Ca^2+^ and heparin binding sites [58].

**Fig. 4:**
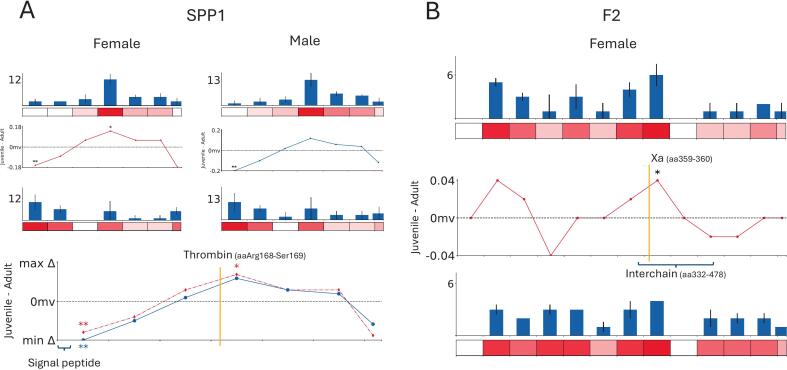
50aa regions of SPP1 (A) and prothrombin (F2) (B) observed to have age-associated changes in PSC abundance. Bar plots present average normalised PSCs per 50aa region, Heatmaps below represent the relative PSC across the protein with bright red representing a high. Line plots show differences in normalised average PSCs (Δavg.PSC) plotted (Δavg.PSC in juvenile rats − Δavg.PSC in adults) against the each 50aa region (significant Δavg.PSC identified by p < 0.05 = *, p < 0.01 = **, p < 0.001 = ***). Published cleavage sites are indicated by a yellow line. (For interpretation of the references to colour in this figure legend, the reader is referred to the web version of this article.)

SPP1 exhibited sex-conserved PSC differences across the protein (Fig. 4A), with one N-terminal 50aa region showing significantly lower in juvenile than adult and another in the centre of the protein, corresponding to the thrombin cleaving domain, which was significantly higher in females.

The increased PSC abundance within the region containing the thrombin cleaving domain in young rats could be a sign of increased cleavage by thrombin and subsequent SPP1 activation/synthesis which could be suggestive of increased bone remodeling. This could also explain the increase in PSCs as the action of thrombin creating the active state of SPP1 subsequently increases active SPP1 solubility and accessibility to trypsin and consequential indication of SPP1′s temporary action limited by heightened probability of degradation. SPP1′s multiple and sometimes contradictory functions in bone remodeling have been observed [58]. The action of SPP1 is a complex web of systematic antagonistic interactions whereby key players such as osteoclasts, osteoblasts, mesenchymal stem cells and hydroxyapatite all seem to interact with SPP1. They do this via either the integrin binding on the N-terminal peptide or the heparin binding domain on the C-terminal peptide. This makes it difficult to pinpoint whether SPP1 is promoting bone growth or resorption but does equally suggest the occurrence of remodeling.

In prothrombin also known by coagulation factor II (F2) only one central 50aa region was found to have significantly greater PSC in female juvenile rats (Fig. 4B). This region contains one of the two Xa factor cleavage sites (aa359–360), that activates thrombin. The region is also located within the interchain found between light and heavy chains and exists as a peptidase S1 domain. The increased PSC abundance in juvenile rats at the Xa cleavage site responsible for thrombin activation corroborates our interpretation of increased bone remodeling potentially via the cleavage of SPP1′s domain by active thrombin. This provides evidence for greater remodeling in developing juveniles compared to adult rats.

If we evaluate the quantity and distribution of peptides uniquely identified in juvenile or adult samples, we see unique peptides identified across the breadth of SPP1 and F2. Interestingly that in SPP1 unique peptide sequences are only identified in juvenile samples compared to F2 where adult samples identified a greater number of unique peptides sequences. Considering the synergistic nature of cleaving SPP1 and F2 in their activity this inverse trend is surprising.

## Age-associated apolipoprotein structural differences reflect changes in lipid composition and dynamics in bone tissue

Apolipoprotein E’s (APOE) primary known role is in the associating with lipid particles, specifically with the purpose of binding and removal/cellular uptake of high-density lipoproteins (HDLs) [59]. Lipids are important part of healthy bone remodeling due to their function in the transport of key nutrients for mineralisation such as vitamins D and K, they also impact the vascularisation bone tissue and the permeability of the bone ECM [59].

**Fig. 5:**
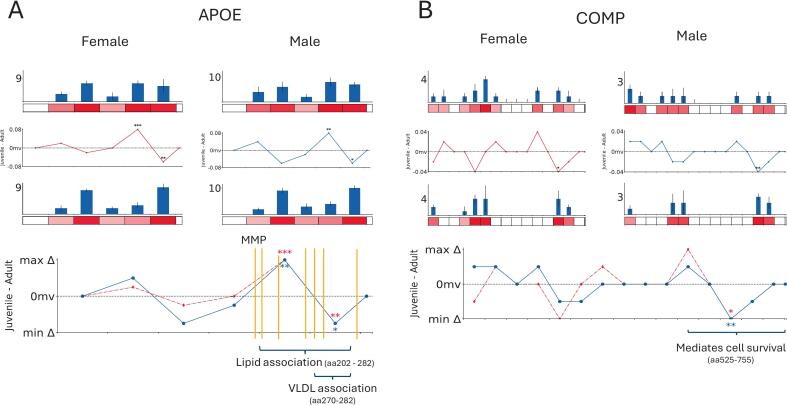
50aa regions of APOE observed to have age-associated differences in PSC abundance. Bar plots represent average normalised PSCs per 50aa region, heatmaps below represent the relative PSC across the protein with bright red representing high. Line plots show differences in normalised average PSC (Δavg.PSC) plotted (Δavg.PSC in juvinile rats − Δavg.PSC in adult) against each 50aa region. Regions with significant differences are indicated (p < 0.05 = *, p < 0.01 = **, p < 0.001 = ***). MMP cleavage sites highlighted by yellow lines. A) Two 50aa regions of significant age-associated difference in Δavg.PSC were identified in APOE, one within the indicated lipid association region and another within the VLDL association region. B) One region of age-associated difference in Δavg.PSC identified within the region of COMP associated with mediating cell survival. (For interpretation of the references to colour in this figure legend, the reader is referred to the web version of this article.)

APOE showed sex-conserved differences across its protein structure, two adjacent regions on the C-terminal side harbouring statistical differences (Fig. 5), the first being higher in juvenile and the other region being lower. The more N-terminal 50aa region lies within the lipid-binding and lipoprotein associated region whereas the other within the very low-density lipoprotein (VLDL) associated domain. We see the VLDL associated domain sequence (RIKGWFEPLVEDM) occur as a specific MS-identified peptide which contributes to the significant difference observed. It is notable that the two regions of both lie close to multiple published MMP14 cleavage sites which suggests age-dependent cleavage potentially by MMPs. This could explain the differences in tryptic peptides abundance within these regions as MMP cleavage creates more soluble fragments accessible to trypsin. Some research shows the cleavage of APOE by MMP14 causes a loss of function [60] which may lead to an accumulation of lipids in the ECM of bone [59]. It is worth noting that PTM analysis highlighted significant statistical increases in the average (median) methionine oxidation (male and female rats) and deamidation (male rats) accumulation (median normalised against total protein abundance) within the region of significantly greater PSC in adults (Supplementary Fig. S5) although these differences were low in number which limits their impact greatly. Further investigating the APOE conformation could potentially account for the different cleavage susceptibility at these two sites in aging. PLF also found age-associated differences in APOA1 and APOA2 (Supplementary Fig. S6). These results reveal a potential role of apolipoproteins in osteogenesis, through potential cleavage and/or interactions with LDL and VLDL, which may be worth pursuing in future.

Cartilage oligomeric matrix protein (COMP) also known as thrombospondin-5 (TSP-5) is an important glycoprotein regulator of bone ECM stability and growth. It’s role in the signalling of collagen synthesis by chondrocytes makes COMP a vital protein for skeletal development. The presence of COMP is a strong indicator of collagen turnover, and defects due to mutations have been associated with the development of several inflammatory conditions like osteoarthritis in bone tissue [60], [61].

Sex-conserved PSC significant differences were seen near the C-terminal end of the protein (Fig. 5B) with higher abundance in adult than juvenile samples. This 50aa region lies within a larger region that mediates cell survival, the induction of the inhibitor of apoptosis family of survival proteins and the thrombospondin (TSP) domain. This attachment region binds to chondrocytes and signals antiapoptotic signalling pathways [60]. Age-specific changes within this region could impact on the ability of COMP to regulate these antiapoptotic signals [62], [63], [64] and therefore wider osteogenesis in juvenile to adult development. Similar results were previously discovered in COMP protein in mouse lung and human intervertebral disc tissue [34] within similarly localised regions however in mouse lung the significant peptide spectral count differences were observed closer to the N-terminal region of COMP. Structure-associated differences in PSC abundance were also seen within wider ECM regulators such as lumican and MMP13 (Supplementary Fig. S6). Lumican has been shown via *in-vitro* experiments to dose dependently reduce bone resorption by inhibiting pathways involved in osteoclastogenesis [65]. MMP13 harboured changes within its propeptide activation region suggesting a change in activity in juvenile rats compared to adults which may reflect the higher rates of remodeling seen in juvenile development. It’s involvement in collagen degradation [45] also links MMP13 to the changes observed in COL1A1 and COL1A2 (Fig. 3).

## Conclusion

This study demonstrates the utility of PLF as a screening tool to identify biomarker candidates of bone aging between juvenile and adult rats within a pre-published, publicly available dataset. This study highlights the importance of fibrillar collagen remodeling and potential new roles for osteopontin, thrombin, apolipoproteins and wider ECM regulators such as COMP. Future related studies should aim to increase the number of samples per age group, include older groups to build a cohesive proteomic narrative of aging in the bone ECM, but could do so using a targeted proteomic approach selecting specific peptides of this study as markers for more accurate assessment of relative abundances. The findings of this study (summarised in Supplementary Table S5) should also be further assessed through *in-vitro* and *in-vivo* experiments that seek to not only validate the trends seen in PLF but also to explore the impact that validated differences have on both protein conformation and folding. Furthermore, experiments exploring the impact of the differences seen in PTMs on protein conformation and *vice versa* would help improve our understanding of age-associated changes in bone that should be later confirmed in humans. Direct interrogation of higher order structures and impact of PTMs found in and around the regions of interest of these identified biomarker candidates could also be highly valuable for confirming changes observed in this proteomic screen. Lastly, given the vastly increasing amounts of proteomic data being made publicly available, this study exemplifies the potential for how novel tools such as PLF could be used to further interrogate a wide range of datasets beyond their initial published studies.

## CRediT authorship contribution statement

**Jacob Tudor:** Writing – review & editing, Writing – original draft, Visualization, Validation, Investigation, Formal analysis. **Alexander Eckersley:** Writing – review & editing, Supervision, Software, Resources, Methodology, Funding acquisition. **Michael Buckley:** Writing – review & editing, Supervision, Project administration, Data curation, Conceptualization.

## Declaration of competing interest

The authors have no financial or personal relationships with other people or organizations that could inappropriately influence or bias this work.

## Data Availability

Although the original raw data is presented elsewhere, the Scaffold-based interpretations have been made available on the FigShare: https://doi.org/10.6084/m9.figshare.30053332; additional information collated in Git repository: https://github.com/jacobtudor/University-of-Manchester-Bioinformatics-Research-Project-2-supplementary-material-JacobTudor.git.
