# Supplementary material for: Revealing sex-specific changes across protein structure in the aging bone extracellular matrix
Source: Matrix Biol Plus. 2025 Dec 25;29:100189. doi: 10.1016/j.mbplus.2025.100189 (PMC12813218; doi:10.1016/j.mbplus.2025.100189)
Supplement: Supplementary Data 1 [file mmc1.doc]

# Supplementary Material

**Table S1: Shows average statistics for peptide modifications identified using PLF at varying levels of stringency with total proteins/peptides (average (avg) peptide spectral count difference (diff) > 1 & p < 0.05) being the proteins/peptides and ultimately containing significant regional differences in peptide spectral counts between juvenile and adult rats**.

| **PLF male dataset statistics** | **Male Number** | **Female Number** |
| --- | --- | --- |
| Total proteins | 109 | 100 |
| Total peptides | 1287 | 1174 |
| Total significant peptides (p < 0.05) | 195 | 155 |
| Total significant proteins (p < 0.05) | 109 | 100 |
| Total peptides (avg peptide spectral count > 0) | 196 | 224 |
| Total proteins (avg peptide spectral count > 0) | 43 | 43 |
| Total peptides  (avg peptide spectral count > 0 & p < 0.05) | 48 | 46 |
| Total proteins  (avg peptide spectral count > 0 & p < 0.05) | 29 | 34 |
| Total peptides (avg peptide spectral count diff > 1) | 70 | 92 |
| Total proteins (avg peptide spectral count diff > 1) | 20 | 27 |
| Total peptides  (avg peptide spectral count diff > 1 & p < 0.05) | 34 | 36 |
| Total proteins  (avg peptide spectral count diff > 1 & p < 0.05) | 20 | 25 |
| Total unique proteins  (avg peptide spectral count diff > 1 & p < 0.05) | 31 | |

**Table S2: Shows the number of unfiltered peptide spectral counts attributed per protein by Mascot MS/MS ion following search per juvenile and adult samples “FZ” and “JA” representing juvenile and adult specimens respectively. Sex identified by “_M” or “_F” for male and female samples, respectively.**

|  | Samples | | | | | | | | | | | | |
| --- | --- | --- | --- | --- | --- | --- | --- | --- | --- | --- | --- | --- | --- |
| Proteins | FZ1_F (RAT1) | FZ2_F (RAT2) | FZ3_F (RAT3) | FZ4_M (RAT4) | FZ5_M (RAT5) | FZ6_M (RAT6) | JA1_M (RAT7) | JA2_F (RAT8) | JA3_M (RAT9) | JA4_M (RAT10) | JA5_F (RAT11) | JA6_F (RAT12) | Total |
| APOE | 13 | 31 | 14 | 23 | 19 | 23 | 36 | 29 | 29 | 36 | 36 | 30 | 795 |
| COL1A1 | 345 | 381 | 351 | 328 | 238 | 371 | 308 | 389 | 389 | 340 | 340 | 463 | 9781 |
| COL1A2 | 152 | 193 | 149 | 146 | 112 | 151 | 178 | 254 | 254 | 230 | 230 | 306 | 5544 |
| COL2A1 | 114 | 137 | 87 | 108 | 70 | 132 | 70 | 72 | 72 | 84 | 84 | 86 | 2497 |
| COMP | 15 | 20 | 7 | 9 | 3 | 11 | 5 | 8 | 8 | 4 | 4 | 5 | 214 |
| F2 | 9 | 13 | 14 | 10 | 6 | 11 | 21 | 33 | 33 | 30 | 30 | 27 | 567 |
| SPP1 | 14 | 40 | 28 | 33 | 9 | 45 | 5 | 3 | 3 | 8 | 8 | 10 | 367 |
| Total | 662 | 815 | 650 | 657 | 457 | 744 | 623 | 788 | 788 | 732 | 732 | 927 |  |

**Table S3: Shows the number of peptide spectral counts attributed per protein by Mascot MS/MS ion following filtering for exclusive peptide spectral counts and FDR < 0.05 search per juvenile and adult samples “FZ” and “JA” representing juvenile and adult specimens respectively. Sex is identified by “_M” or “_F” for male and female samples respectively. A) shows the peptide spectral counts found within female samples. B) shows the peptide spectral counts found within male samples**

A

|  | **Samples** | | | | | | |
| --- | --- | --- | --- | --- | --- | --- | --- |
| **Protein** | FZ1_F (RAT1) | FZ2_F (RAT2) | FZ3_F (RAT3) | JA2_F (RAT8) | JA5_F (RAT11) | JA6_F (RAT12) | **Total** |
| APOE | 13 | 31 | 14 | 29 | 37 | 30 | 154 |
| COL1A1 | 482 | 522 | 486 | 533 | 463 | 632 | 3118 |
| COL1A2 | 199 | 261 | 194 | 339 | 315 | 411 | 1719 |
| COL2A1 | 149 | 192 | 121 | 101 | 113 | 114 | 790 |
| COMP | 21 | 27 | 9 | 12 | 5 | 8 | 82 |
| SPP1 | 20 | 48 | 35 | 4 | 12 | 12 | 131 |
| F2 | 11 | 18 | 20 | 41 | 39 | 34 | 163 |
| **Total** | 895 | 1099 | 879 | 1059 | 984 | 1241 |  |

B

|  | **Samples** | | | | | | |
| --- | --- | --- | --- | --- | --- | --- | --- |
| Protein | FZ4_M (RAT4) | FZ5_M (RAT5) | FZ6_M (RAT6) | JA1_M (RAT7) | JA3_M (RAT9) | JA4_M (RAT10) | total |
| APOE | 23 | 19 | 23 | 36 | 29 | 37 | 167 |
| COL1A2 | 191 | 160 | 203 | 262 | 339 | 315 | 1470 |
| COL2A1 | 147 | 94 | 178 | 95 | 101 | 113 | 728 |
| COMP | 12 | 3 | 15 | 6 | 12 | 5 | 53 |
| SPP1 | 46 | 12 | 61 | 8 | 4 | 12 | 143 |
| **Total** | 419 | 288 | 480 | 407 | 485 | 482 |  |

**Table S4**: Shows the number of unique peptides sequences identified only in adult/juvenile and, the distribution of their identification across male and female samples, that contributed to PLF peptide spectral matches for each protein.

|  |  |  | **Sex** | |
| --- | --- | --- | --- | --- |
| *Protein* | Age | Total | Female | Male |
| *APOE* | Adult | 12 | 4 | 8 |
|  | Juvenile | 3 | 2 | 1 |
| *COL1A1* | Adult | 19 | 19 | 0 |
|  | Juvenile | 4 | 4 | 0 |
| *COL1A2* | Adult | 41 | 24 | 17 |
|  | Juvenile | 10 | 6 | 4 |
| *COL2A1* | Adult | 17 | 11 | 6 |
|  | Juvenile | 33 | 11 | 22 |
| *COMP* | Adult | 3 | 2 | 1 |
|  | Juvenile | 14 | 3 | 11 |
| *F2* | Adult | 12 | 12 | 0 |
|  | Juvenile | 2 | 2 | 0 |
| *SPP1* | Juvenile | 11 | 11 | 0 |


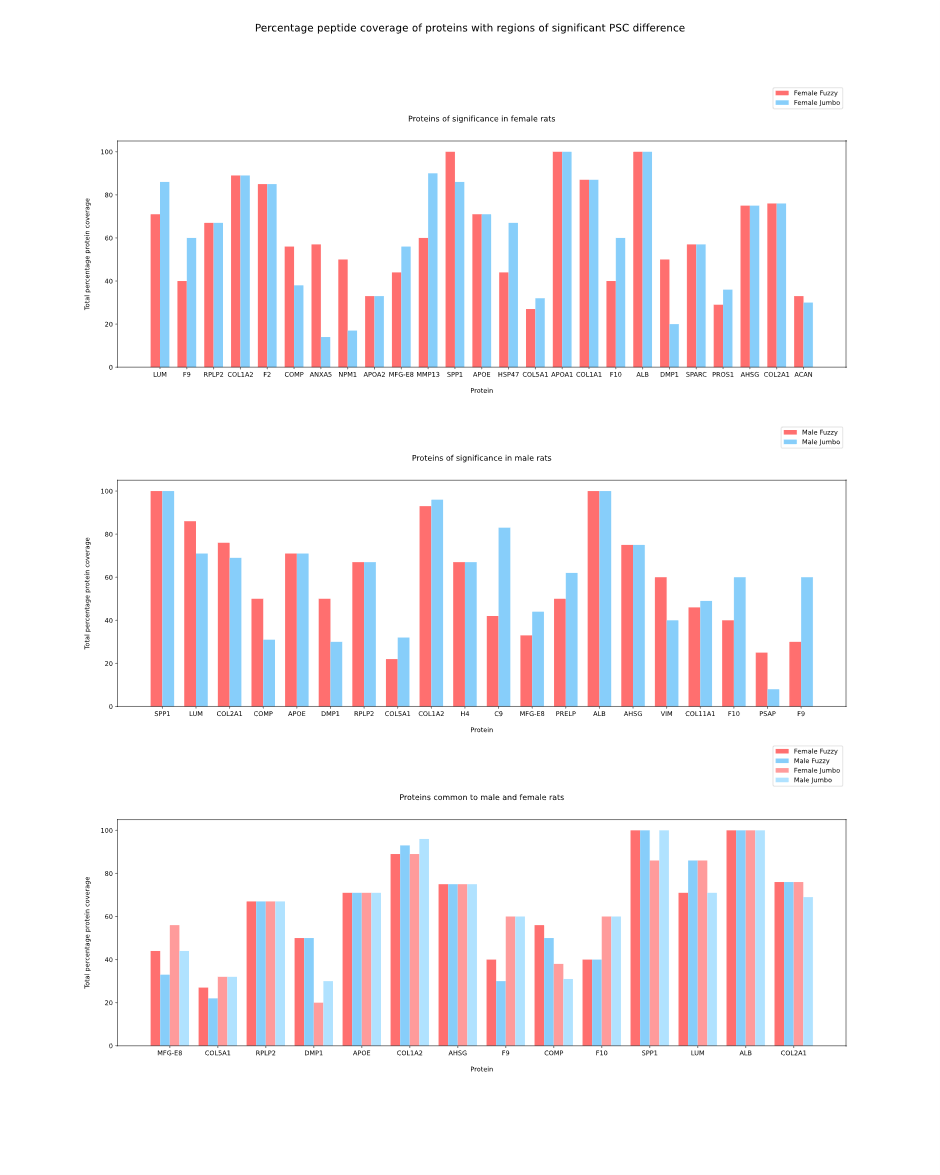


Figure S1 - Bar plot showing the total percentage peptide coverage of each protein deemed to have significant PLF identified peptide modification differences between fuzzy and jumbo rats. Coverage in female rats shown in red and coverage seen in male rat data seen in blue. Juvenile (fuzzy) rats’ data shown in brighter colour while adult (jumbo) rat data is shown in lighter colour. A) Female rats. B) Male rat. C) Common to both male and female rats. Repeated searches for PTMs and uniquely identified peptides (from Table S3) across PLF-identified regions of age-associated structural differences and the associated custom built scripts are provided with additional information collated in Git repository: [**https://github.com/jacobtudor/University-of-Manchester-Bioinformatics-Research-Project-2-supplementary-material-JacobTudor.git**](https://github.com/jacobtudor/University-of-Manchester-Bioinformatics-Research-Project-2-supplementary-material-JacobTudor.git)


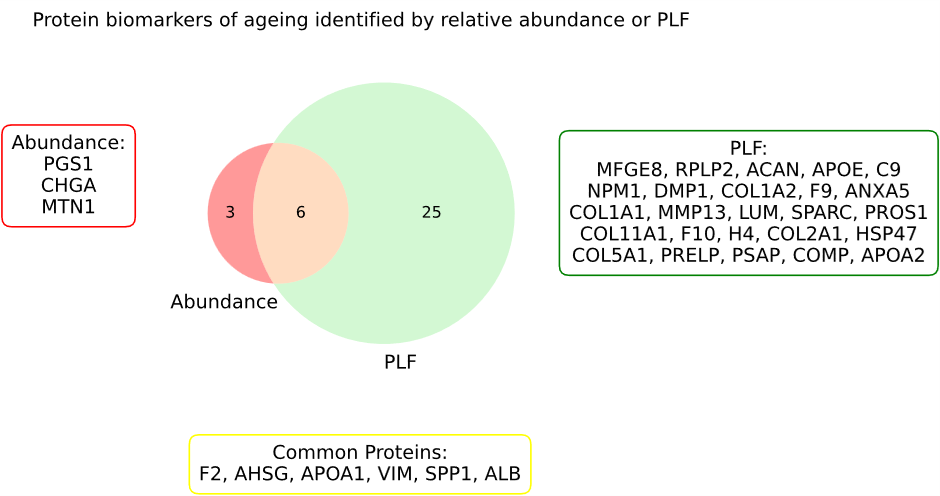


**Figure S2: Venn plot displaying protein biomarkers of ageing in rat bone ECM identified by relative abundance (LC-MS/MS) in Johnston and Buckley (2021) and by Peptide Location Fingerprinting (PLF). Three potential protein biomarkers of ageing identified using relative abundance data only. 25 were identified using the results of PLF derived from the relative abundance data. Six proteins identified as potential biomarkers of ageing utilising relative abundance data or PLF.**


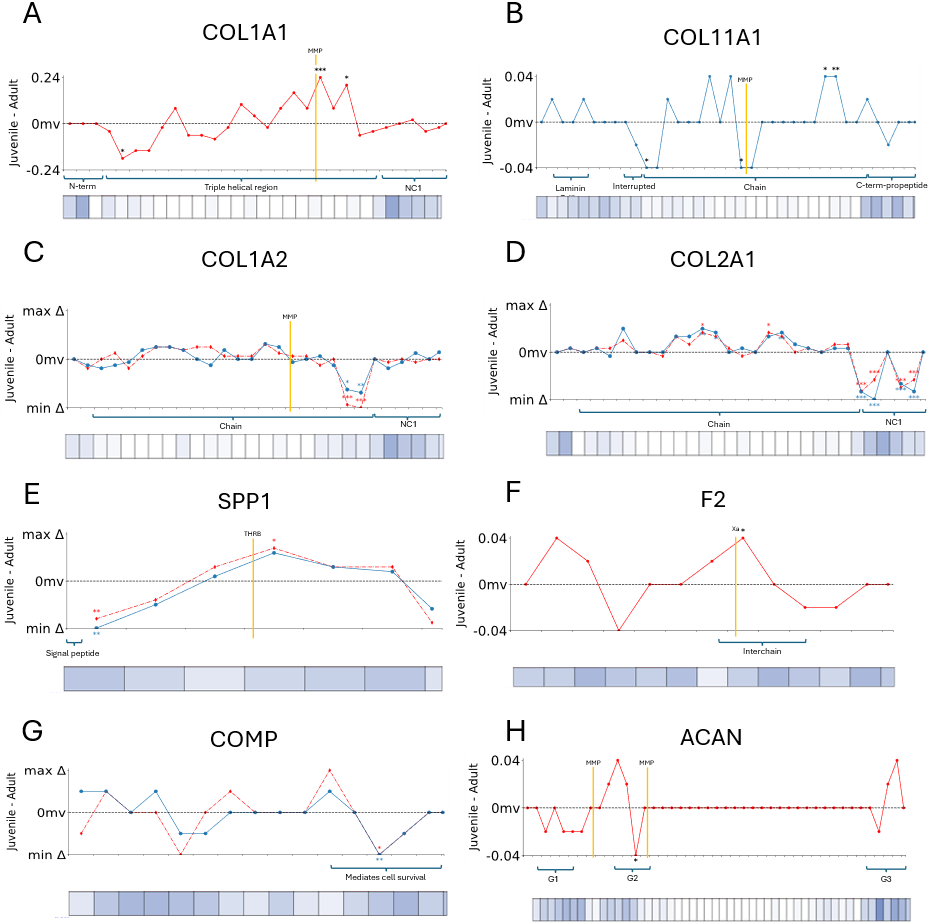


**Figure S3: Difference in normalised average PSC (∆avg.PSC) plotted (∆avg.PSC in juvenile (fuzzy) rats [1-2 weeks old] - ∆avg.PSC adult (jumbo) [10 weeks – 6 months old]) in male (blue) and female (red) rats against the mapped 50aa peptide region along the MS/MS spectrum identified proteins. Levels of significance identified by p < 0.05 = *, p < 0.01 = **, p < 0.001 = ***. Published cleavage sites indicated with yellow. Heatmap shows the estimated ROS susceptibility of the human equivalent of proteins per 50aa region as defined by the Manchester Proteome Susceptibility Calculator (Dark blue representing high susceptibility to ROS).**


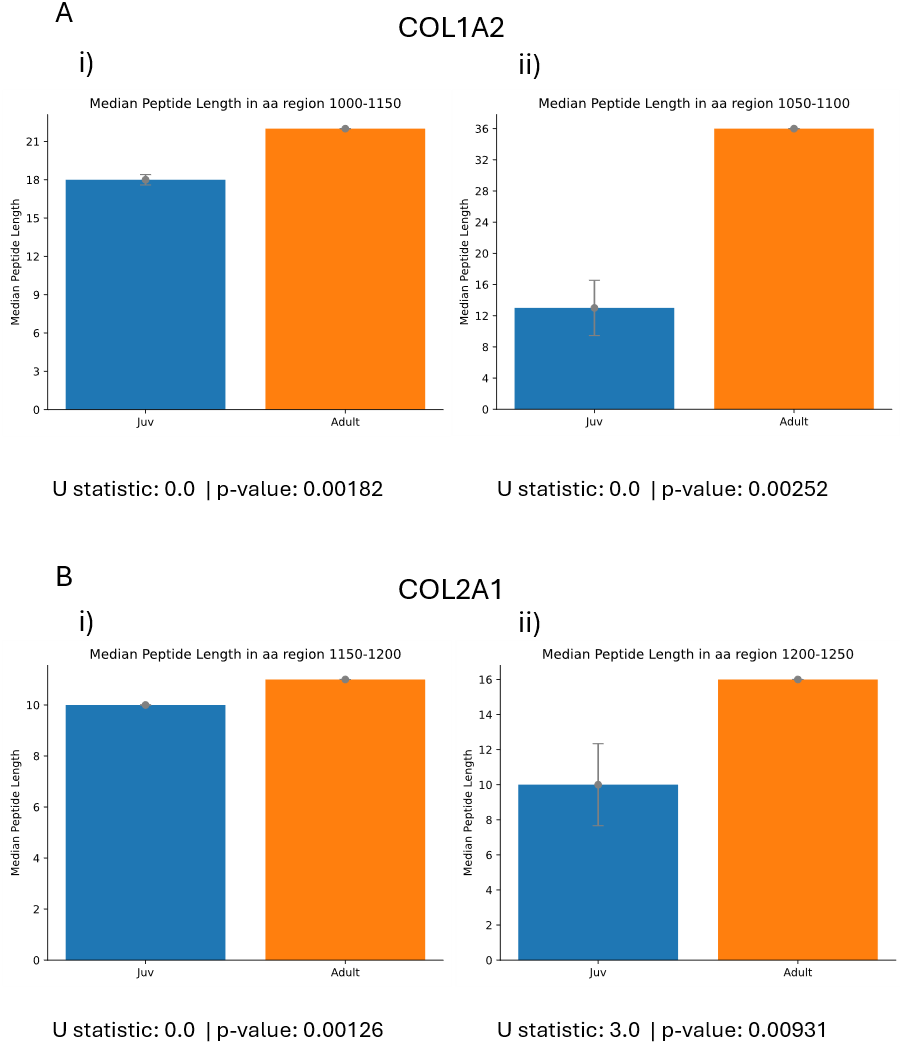


**Figure S4: Median peptide length identified within amino acid regions containing significant differences in peptide spectral counts between juvenile and adult, (male and female) rats specimens. Median values present average peptide length across all replicates within juvenile (6 samples) and adult replicates (6 samples). Mann-Whitney U test (p = 3s.f) performed upon calculated median peptide length of each replicate per age group. Error bars indicate standard deviation between median peptide length of each experimental replicate. In A) i) COL1A2 1000-1050 we only see a change in median peptide length of ~3aa and ii) 1050-1100 with twice the median peptide length within region 1050-1100 being approximately twice as long in adults than in juvenile rats across. In COL2A1 B) i) very little biological significance observed in between juvenile and adult rats within region 1150-1200. Region 1200-1250 however shows a median difference in peptide length of approximately 6aa. These differences in median peptide length indicate increased susceptibility to tryptic cleavage in juvenile rats compared to adult rats within regions of significantly higher peptide spectral count in adult rats.**


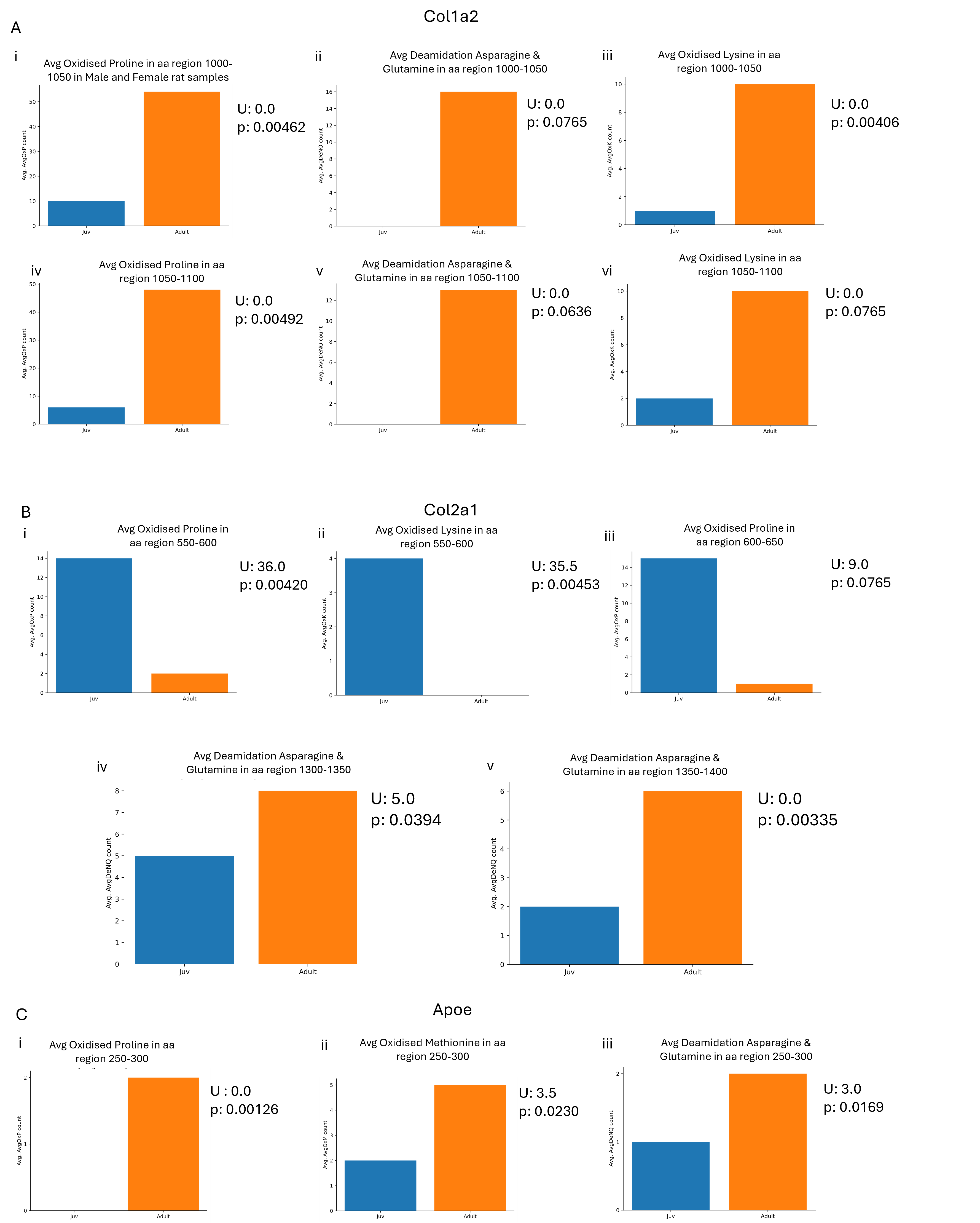


**Figure S5: Mean number of oxidised prolines or methionines and deamidated asparagine and glutamines detected with significant differences in quantity between juvenile and adult rat bone samples in proteins A) COL1A2, B) COL2A1, C) APOE, in regions detected to contain significant differences in peptide spectral count between juvenile and adult rats. Mann-Whitney U Test expressed statistically significant differences in the number of oxidised prolines, methionines and deamidation of asparagine and glutamines in these regions providing indicators of oxidative stress (oxidation of methionine) and increased susceptibility to tryptic cleavage.**


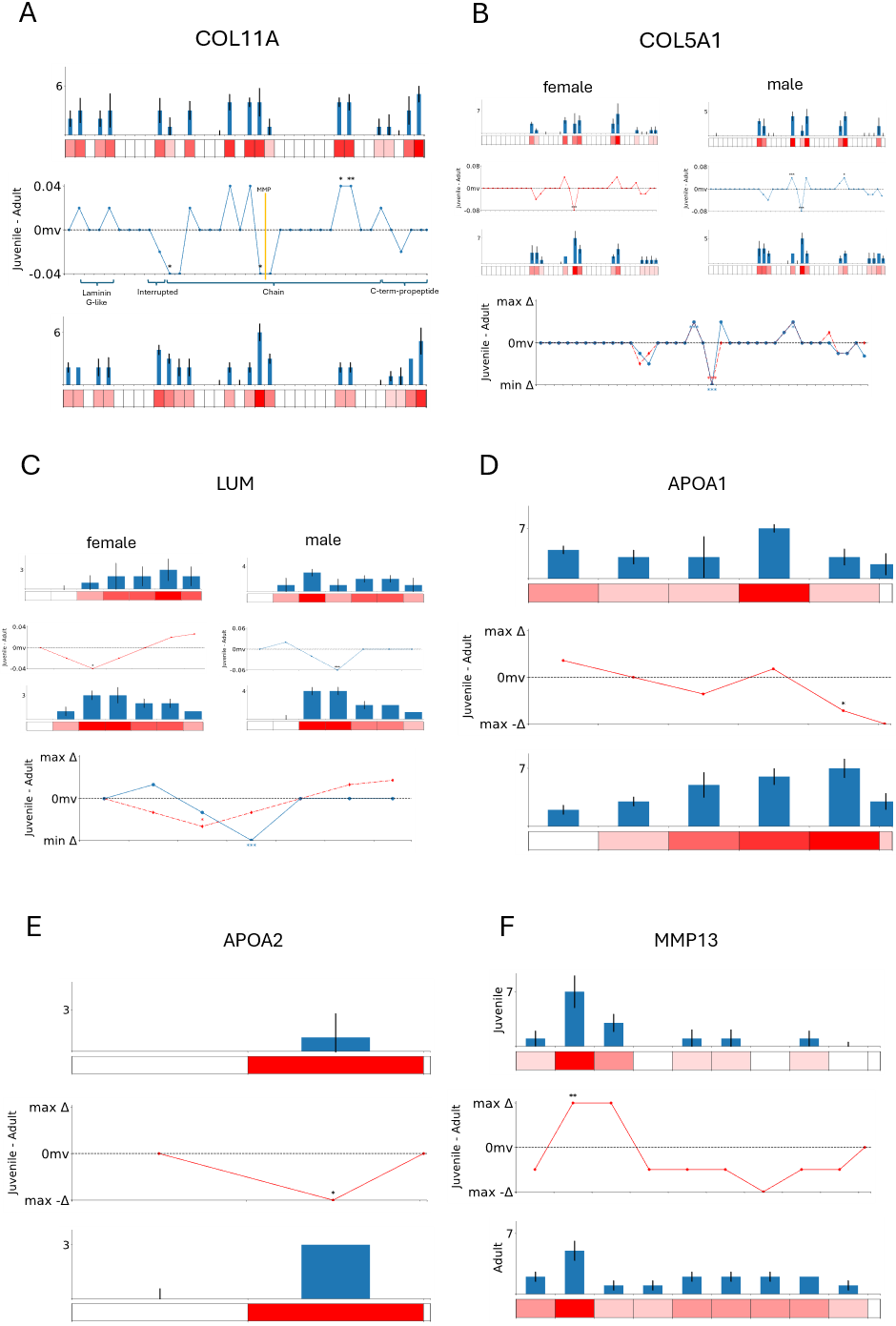


**Figure S6: Bone extracellular matrix proteins with 50aa regions observed to have age-associated changes in average normalised PSC. Barplots represent average normalised peptide spectral count per 50aa region, heatmap below representing the relative PSC across the protein, bright red representing a high relative PSC. Barplots only show male or female samples if a region of significant change in average normalised PSC (∆avg.PSC) was detected in that dataset. Difference in normalised average PSC (∆avg.PSC) plotted (∆avg.PSC in fuzzy rats [1-2 weeks old] - ∆avg.PSC jumbo [10 weeks – 6 months old]) against the mapped 50aa peptide region along the MS/MS spectrum identified proteins. Levels of significance identified by p < 0.05 = *, p < 0.01 = **, p < 0.001 = ***. MMP cleavage site heatmap (red) mapped beneath plot separated into 50aa regions for human equivalent of the rat protein. Published cleavage sites indicated with yellow. A) COL11A1 harboured four regions of significant age-associated difference One near the start of the tiple helical chain, another at a known MMP9 cleavage site and two upstream of the C-terminal propeptide. B) Shows 3 significant regions with age-associated changes in COL5A1. C) Shows 2 regions of age-associated changes in protein structure in lumican. D/E) Shows 1 region of significant age-associated change in protein structure in APOA1 and APOA2. F) Shows 1 region of significant age-associated change in protein structure in MMP13.**
